# Digital health education and self-management support for adults with heart failure: a best-evidence summary of review-level evidence

**DOI:** 10.3389/fpubh.2026.1926335

**Published:** 2026-09-14

**Authors:** Huan Dong, Zhankun Jia, Xia Wang, Guiling Yang

**Affiliations:** Aerospace Center Hospital, Beijing, China

**Keywords:** digital health, evidence summary, evidence-based nursing, health education, heart failure, mHealth, self-management, telemonitoring

## Abstract

Heart failure (HF) is a major public-health burden, and sustained patient education and self-management support are central to long-term care. Digital health technologies provide additional channels for education, monitoring and post-discharge follow-up, but review-level evidence is heterogeneous across intervention models, intensity, feedback mechanisms and outcomes. This study aimed to synthesise review-level evidence on digital health education and digitally supported self-management for adults with HF and to develop practice-oriented evidence statements. We conducted a narrative best-evidence synthesis of review-level evidence, informed by JBI evidence-summary principles and reported according to PRISMA 2020 where applicable. The protocol was registered with the Fudan University Centre for Evidence-Based Nursing (ES20259435). Eight English and Chinese bibliographic databases were searched from inception to 1 December 2025. Eligible evidence included systematic reviews, meta-analyses, network meta-analyses, umbrella reviews, systematic metareviews and scoping reviews indexed in the searched databases. Two reviewers independently screened sources, extracted data and appraised methodological quality using AMSTAR 2 or PRISMA-ScR/JBI-informed criteria. Evidence statements were developed using review quality, directness, consistency, feasibility and likely overlap among primary studies; no recommendation grade was assigned. Of 8,698 records identified, 5,331 were screened after de-duplication, 37 full-text reports were assessed and 27 review-level evidence sources were included. Evidence varied substantially in quality, directness and intervention type. The final synthesis generated 27 practice-oriented statements organised into three tiers: essential core components, conditional implementation components, and emerging or outcome-related statements. Review-level evidence suggests that digitally supported education and self-management may complement routine HF care, particularly when linked to structured clinical feedback, clearly assigned responsibility and escalation pathways. However, the evidence statements should be interpreted conditionally because of methodological limitations, indirectness, intervention heterogeneity and likely overlap among reviews.

## 1 Introduction

Heart failure (HF) remains one of the leading global public-health problems. Its prevalence continues to rise with population ageing and improved survival after cardiovascular disease, and it places a substantial burden on health systems and social care. Epidemiological reviews estimate that approximately 64 million people worldwide are affected by HF, and community-based chronic HF continues to have a poor long-term prognosis, with a pooled five-year survival of about 57% (1, 2). The HF STATS 2024 report further indicates that HF-related mortality, healthcare utilisation and economic burden continue to increase (3). After discharge, patients often experience symptom fluctuation, medication adjustment and readmission risk, making continuous in and out-of-hospital management a priority for clinical care and public health.

Structured education and self-management support are central components of long-term HF management. The 2021 ESC guidelines and the 2022 AHA/ACC/HFSA guideline emphasise multidisciplinary HF management programmes, patient education, self-care support, psychosocial support and post-discharge follow-up (4, 5). However, traditional face-to-face education is limited by staff time, unequal access to specialised HF services, patient mobility, health literacy and adherence. These constraints make it difficult to deliver repeated, individualised and sustained education after patients leave the hospital.

Digital health technologies, including mHealth applications, telemonitoring, web portals, WeChat/SMS, interactive voice response, wearable devices and emerging AI-based tools, may extend education and self-management support beyond hospital-based care. These tools can support disease education, symptom recognition, medication reminders, dietary and fluid guidance, exercise support, weight and blood-pressure monitoring, alerts and professional feedback. The World Health Organization has emphasised the role of digital technologies in improving health-service accessibility, efficiency and equity (6).

Existing reviews suggest potential benefits of mHealth education, structured telephone support and remote monitoring in HF, but the evidence is fragmented (7–10). Reviews differ in population, technology, education content, feedback mechanism, intensity, comparator and outcome. Some sources focus directly on HF education, whereas others address broader eHealth, telemonitoring or chronic-disease self-management. AI and large-language-model tools are also emerging, but their accuracy, safety and clinical impact in HF education remain insufficiently validated (11–14).

Therefore, this study synthesised review-level evidence on digital education and digitally supported self-management for adults with HF into practice-oriented evidence statements. The aim was not to re-estimate intervention effects, but to clarify implementation-relevant questions: who should deliver digital education, which patients should be prioritised, how platforms and components should be selected, what educational content is essential, how monitoring and feedback should be organised, how intervention dose should be interpreted and how equity, privacy, safety and AI-related risks should be addressed.

## 2 Methods

### 2.1 Study design and reporting

This study was conducted as a narrative best-evidence synthesis of review-level evidence. It was informed by JBI evidence-summary principles, including systematic retrieval, critical appraisal, evidence extraction, grading and transformation of evidence into practice-oriented statements (15). It was not conducted as a formal JBI Evidence Summary product or as a quantitative overview of reviews. PRISMA 2020 was used to guide transparent reporting of search, selection and synthesis procedures because the review question involved systematic retrieval and selection of evidence sources; overview-specific concerns such as directness, overlap and review quality were additionally addressed in the methods, results and supplementary evidence map (16). The protocol was registered with the Fudan University Centre for Evidence-Based Nursing (registration No. ES20259435). Quantitative pooling was not performed because the evidence sources differed substantially in design, intervention components, comparators, outcomes and analytic methods.

### 2.2 Question development (PIPOST)

Using the PIPOST framework, the population was adults (> = 18 years) with HF of any phenotype (HFrEF, HFmrEF or HFpEF) and any NYHA class, including hospitalised, post-discharge, ambulatory and community patients. The intervention was digital health education or digitally delivered self-management/self-care support, including mobile applications, SMS/WeChat or other messaging platforms, web portals, telephone or interactive voice response, wearable devices, remote monitoring systems and AI-related tools when they were used for education or self-management support. Professionals included nurses, physicians, pharmacists and other multidisciplinary team members. Outcomes included HF knowledge, self-care/self-management behaviour, self-efficacy, medication adherence, health-related quality of life, psychological outcomes, mortality, hospitalisation/readmission, emergency department visits, satisfaction and acceptability. Settings included hospital, community and home/post-discharge care. Eligible evidence types were published review-level evidence indexed in bibliographic databases, including systematic reviews, meta-analyses, network meta-analyses, umbrella reviews, systematic metareviews and scoping reviews.

### 2.3 Search strategy

Eight bibliographic databases were searched from inception to 1 December 2025: PubMed, Web of Science, Cochrane Library, Embase, CNKI, Wanfang, VIP/CQVIP and SinoMed. Search terms combined three concept blocks: HF; digital health or technology; and education or self-management. Subject headings and free-text terms were combined in English databases, and corresponding Chinese subject terms and keywords were used in Chinese databases. The formal search was limited to bibliographic databases. We did not conduct a systematic search of guideline repositories, professional society websites, JBI evidence summaries, WHO resources or other grey-literature sources. This synthesis should therefore be interpreted as a synthesis of indexed review-level evidence rather than as a comprehensive guideline or grey-literature synthesis. Full database search strings, search dates and retrieval counts are provided in Supplementary material S1. Reports available online by the search cutoff were eligible even if assigned to a later print issue.

### 2.4 Eligibility criteria

Inclusion criteria were: (1) population, intervention and content consistent with the PIPOST framework; (2) eligible review-level evidence indexed in the searched bibliographic databases; (3) Chinese or English language; and (4) full text available. Exclusion criteria were: (1) populations other than adults with HF or evidence in which HF findings could not be separated or judged relevant; (2) interventions not digitally delivered or not related to education, self-management, self-care support, monitoring/feedback or patient-provider communication; (3) primary studies, protocols, conference abstracts, editorials, letters, non-systematic narrative reviews or reports without sufficient systematic-review methods; (4) duplicate or less complete reports of the same review; and (5) full text unavailable.

### 2.5 Study selection and data management

Records were imported into reference-management software and de-duplicated. Two reviewers (HD and XW) independently screened titles/abstracts and then full texts. Disagreements were resolved through discussion, with GY consulted when consensus was required. A total of 8,698 records were identified; after removing 3,367 duplicates, 5,331 records were screened by title and abstract, 5,294 were excluded and 37 reports were sought and retrieved. All 37 reports were assessed for eligibility, and ten were excluded with reasons: eight conference abstracts, abstract-only, duplicate or incomplete reports, one report with an irrelevant digital intervention/content focus, and one non-systematic narrative review. Twenty-seven review-level evidence sources were included in the synthesis (Figure 1).

**Figure 1:**
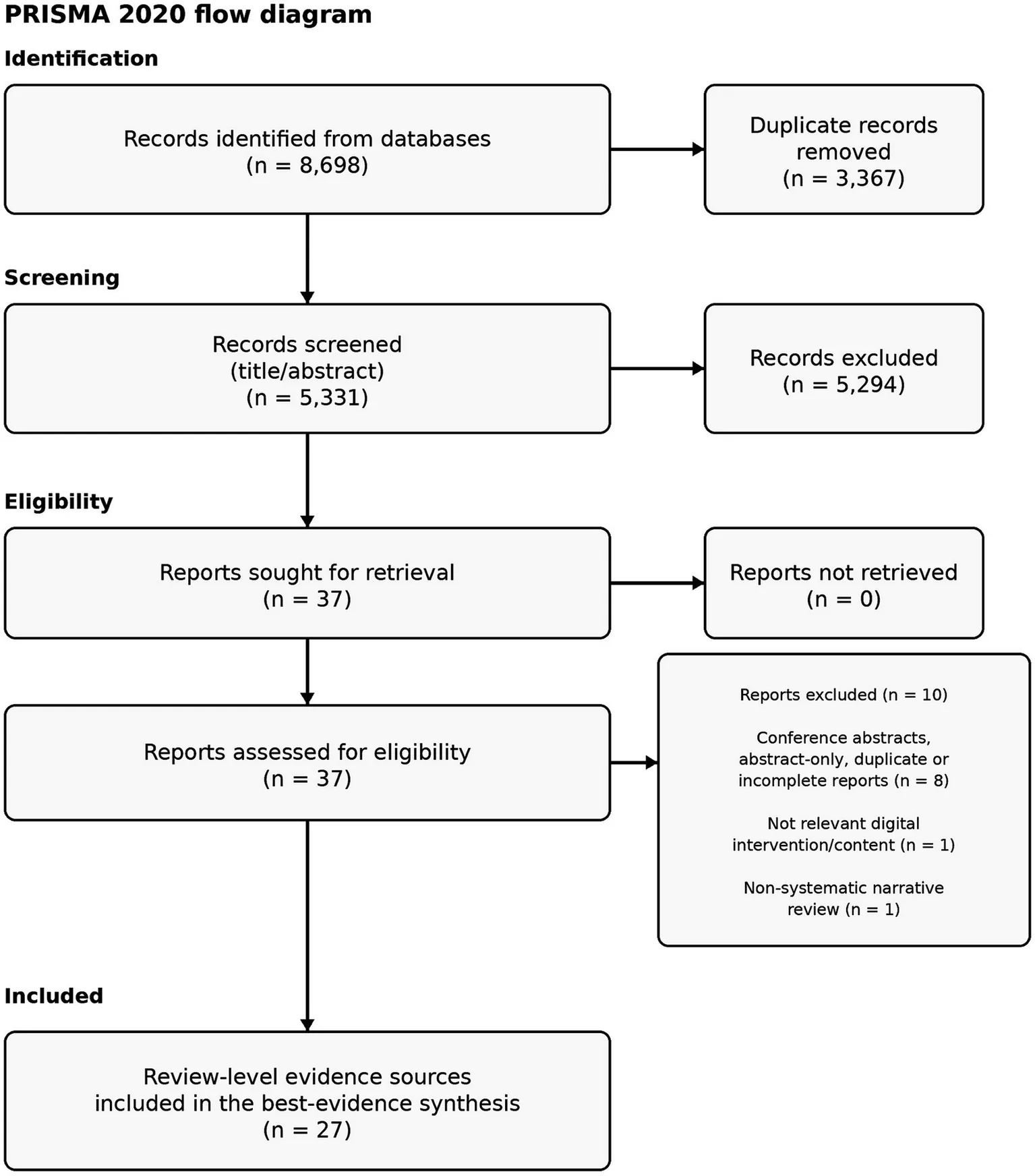
PRISMA 2020 flow diagram of study selection.

### 2.6 Methodological quality appraisal

Two reviewers (HD and XW) independently appraised methodological quality. Systematic reviews, meta-analyses, the network meta-analysis, umbrella review and systematic metareview were appraised with AMSTAR 2 (17). Scoping reviews were not rated with AMSTAR 2 and were assessed descriptively using PRISMA-ScR and JBI-informed criteria. The AMSTAR 2 appraisal considered all 16 items, including critical domains such as protocol registration, adequacy of the search, duplicate study selection/data extraction, excluded-study lists, risk-of-bias assessment, appropriateness of synthesis, interpretation in light of risk of bias and publication bias. A 16-item AMSTAR 2 matrix, together with scoping-review appraisal notes, is provided in Supplementary material S2. Appraisal findings were used to interpret credibility and to downgrade statement strength when evidence was indirect, inconsistent, methodologically weak or likely overlapping.

### 2.7 Evidence statement development and use

Evidence statements were developed from convergent findings across eligible reviews and interpreted using JBI FAME considerations (feasibility, appropriateness, meaningfulness and effectiveness) alongside review quality, directness to adult HF digital education, consistency, applicability, resource implications and likely review overlap. FAME was used as an interpretive framework rather than as a formal recommendation-grading system. No A/B recommendation grade or JBI level was assigned. Statements based on mixed chronic-disease evidence, broad telehealth evidence, low or critically low-confidence reviews, component-level inference or author implementation interpretation were worded conditionally and accompanied by explicit guidance on use.

### 2.8 Evidence extraction, synthesis and overlap handling

Two reviewers independently extracted target population, delivery personnel, platform/modality, education content, monitoring/feedback components, intervention duration and intensity, outcomes, applicability and safety considerations. Evidence was grouped into domains by thematic synthesis. Candidate statements were drafted from convergent findings, checked against supporting reviews and refined through consensus discussion among the review authors. No external expert panel was convened, and the statements should therefore be interpreted as evidence-informed practice statements rather than expert-consensus recommendations. Because many reviews covered similar primary trials, especially in structured telephone support, telemonitoring, mHealth, nurse-led eHealth and RPM, supporting reviews were not counted as fully independent bodies of evidence. A complete corrected covered area calculation could not be performed because several reviews did not report full primary-study lists in extractable form. Instead, a statement-level evidence map was created to indicate likely overlap risk, directness and quality concerns. Overlap risk and indirectness were used to temper statement wording and interpretation (Supplementary material S3).

## 3 Results

### 3.1 Search results and characteristics of included studies

The study selection process is shown in Figure 1. Twenty-seven review-level evidence sources were included in the final synthesis: three Cochrane reviews (7–9), one Bayesian network meta-analysis (11), one umbrella review (12), one systematic metareview (13), one systematic review of AI/large-language-model applications (14), one restricted systematic review (18), two scoping reviews (19, 20) and multiple systematic reviews/meta-analyses of mHealth, eHealth, distance education, nurse-led telecoaching, remote monitoring and integrated care (10, 21–36). The evidence base was heterogeneous. Some sources were directly focused on adult HF and digital education, whereas others addressed broader self-management, telehealth or chronic-disease populations with HF subgroups. Table 1 summarises study-level information, including population/intervention directness, main findings and appraisal rating.

**Table 1:** Study-level characteristics, relevance and appraisal of included review-level evidence.

Ref.	Evidence source/design	Population and directness	Digital intervention relevance	Main outcomes or findings	Appraisal/use
Flodgren et al. (7)	Cochrane SR/MA; 93 trials, 22,047 participants; HF subset 16 trials	Mixed chronic conditions; HF subset; indirect for education	Interactive telemedicine/telemonitoring	Health service outcomes; broad telemedicine effects	High; indirect
Allida et al. (8)	Cochrane SR/MA; 5 RCTs, 971 participants	Adult HF; direct	mHealth education	HF knowledge, self-care, self-efficacy, QoL, hospitalisation	High; direct
Inglis et al. (9)	Cochrane SR/MA; 41 RCTs	HF; direct	Structured telephone support/non-invasive telemonitoring	Mortality, HF hospitalisation, QoL	High; direct but telemonitoring broader than education
Bezerra Giordan et al. (10)	Systematic review; 28 studies, 10 RCTs	HF; direct	Self-management mobile apps	App components, usability, self-management outcomes	Low; direct
Li et al. (11)	Bayesian NMA; 21 RCTs	Adult HF NYHA II-IV; direct	eHealth self-management interventions	Comparative effectiveness across eHealth models	Critically low; direct but heterogeneous
da Costa Ferreira Oberfrank et al. (12)	Umbrella review; 44 SRs, 135 studies	Adult CHF; direct/broad	Self-care interventions including telehealth education	Self-care, QoL, hospitalisation-related outcomes	Low; overlap likely
Hanlon et al. (13)	Systematic metareview; 53 SRs; HF subset 9	Mixed long-term conditions; HF subset; indirect	Telehealth self-management	Self-management and health service outcomes	Critically low; indirect
Doyle et al. (14)	Systematic review; 7 observational/methodological studies	Adult HF; direct for AI topic	ChatGPT/LLM applications	Education accuracy/readability; no clinical outcome validation	Critically low; early-stage evidence
Morken et al. (18)	Restricted systematic review; 18 studies, 16 RCTs	Post-discharge HF; direct	eHealth follow-up after hospitalisation	Post-discharge follow-up, self-management, readmission	Low; direct
Chen et al. (19)	Scoping review; 84 studies	CHF; direct/broad	Mobile health management	Monitoring, education, support, cost/sustainability topics	Scoping; mapping not effect certainty
Qi et al. (20)	Scoping review; 17 studies, 6 RCTs	Post-discharge HF; direct	ICT transitional care	Rehospitalisation, adherence, patient support	Scoping; mapping not effect certainty
Yan et al. (21)	Meta-analysis; 14 RCTs, 2,778 participants	CHF; direct	Internet+ nursing intervention	Readmission, QoL, self-care, mortality	Critically low; supportive only
Ding et al. (22)	Meta-analysis; 22 RCTs, 5,149 participants	CHF NYHA II-III; direct	eHealth disease management	QoL, self-care, readmission, mortality	Critically low; supportive only
Zhang N. et al. (23)	SR/MA; 24 RCTs, 3,660 participants	CHF; direct	Nurse-led eHealth	Illness management, self-care, QoL	Critically low; direct but quality concerns
Imanuel Tonapa et al. (24)	SR/MA; 12 RCTs, 1,938 participants	Adult HF NYHA II-IV; direct	Nurse-led telephone coaching	Self-care, QoL, hospitalisation-related outcomes	Critically low; supportive only
Yang et al. (25)	SR/MA; 105 RCTs, 37,607 participants	CHF; direct/broad	Multicomponent integrated care including eHealth	Hospitalisation, mortality, QoL	Critically low; broad intervention
Kitsiou et al. (26)	SR/MA; 16 RCTs, 4,389 participants	Adult HF; direct	mHealth ± monitoring/feedback	HF hospitalisation, mortality, self-management	Critically low; direct but overlap/heterogeneity likely
Inglis et al. (27)	Abridged Cochrane SR/MA; 30 RCTs, 8,323 participants	CHF; direct	Telemonitoring/telephone support	Hospitalisation, mortality, QoL	Low; direct but overlaps with Ref. 9
Zhang J. et al. (28)	Meta-analysis; 13 RCTs, 2,534 participants	Adult HF; direct	mHealth and medication adherence	Medication adherence, readmission, mortality	Critically low; direct
Ni et al. (29)	SR/MA; 24 studies, 2,886 participants	Adult HF; direct	Mobile application interventions	Self-care, QoL, hospitalisation-related outcomes	Low; direct
Sun et al. (30)	SR/MA; 15 RCTs	Chronic/stable HF; direct	Distance education	Self-care, knowledge, QoL	Critically low; direct
Liu et al. (31)	SR/MA; 24 RCTs, 9,634 participants	Adult HF; direct	eHealth self-management	Self-care, QoL, hospitalisation, mortality	Critically low; direct but heterogeneous
De Lathauwer et al. (32)	SR/MA; 41 RCTs, 16,312 participants	Adult HF; direct	Remote patient monitoring	Programme components for hospitalisation/mortality	Critically low; direct; overlap likely
Mouselimis et al. (33)	Systematic review; 17 studies, 11 RCTs	HF; direct	Patient-oriented mHealth	mHealth features and patient outcomes	Critically low; supportive/contextual
Camino Ortega et al. (34)	SR of RCTs; 8 RCTs	Adult HF; direct	Digital therapeutic education + monitoring	Knowledge, readmission, functional/self-care outcomes	Critically low; direct
de Jong et al. (35)	Systematic review; 15 studies	Mixed chronic conditions incl. HF; indirect	Asynchronous patient-provider communication	Health behaviour and outcomes	Critically low; indirect
Azizi et al. (36)	SR of RCTs; 5 RCTs, 870 participants	Rural HF; direct to underserved settings	Telemedicine/RPM/mHealth	Rural HF self-management and service access	Critically low; direct to rural context

This table is a study-level inventory of included review-level evidence. Directness, intervention relevance and appraisal in this table refer to each included review or evidence source, not to individual evidence statements in Table 3.

### 3.2 Methodological quality and appraisal transparency

Among the 25 sources appraised with AMSTAR 2, three were rated high, none moderate, five low and seventeen critically low. The two scoping reviews were assessed descriptively rather than assigned AMSTAR 2 ratings. Under AMSTAR 2, one critical flaw results in low overall confidence and more than one critical flaw results in critically low confidence; a not-reported judgement in a critical domain was treated as not meeting that domain for the overall rating. The most frequent critical limitations were absence of an excluded-study list with reasons and lack of prospective protocol reporting. Incomplete reporting of funding sources for included primary studies and reliance on composite quality scales rather than domain-based risk-of-bias assessment were also common. These weaknesses affected synthesis by lowering confidence in strongly worded statements. Low and critically low-confidence reviews were retained only as supportive or contextual evidence and did not alone justify strong practice statements. Details are provided in Table 2 and Supplementary material S2.

**Table 2:** Overall methodological quality and use in synthesis.

Rating/approach	*n*	References	Use in synthesis
High	3	(7–9)	Used as the most credible evidence but still interpreted according to directness and overlap.
Moderate	0	—	No included AMSTAR 2 appraisal met the overall moderate-confidence definition after critical-domain rules were applied.
Low	5	(10, 12, 15, 24, 26)	Used cautiously; each review had one critical flaw, most commonly no excluded-study list with reasons.
Critically low	17	(11, 13, 14, 18–23, 25, 27–33)	Used mainly as contextual or supportive evidence; not used alone to justify strong practice statements.
Scoping review	2	(16, 17)	Used for implementation mapping rather than effect certainty.

### 3.3 Directness and overlap considerations

Directness varied across the evidence base. Evidence was considered most direct when the source focused on adults with HF and included education or self-management support delivered through digital means. Evidence was considered indirect when HF was only a subgroup of a mixed chronic-disease review, when the digital intervention was broader telehealth or monitoring without a clearly separable education component, or when evidence related primarily to implementation/safety rather than patient outcomes. Overlap among primary studies was likely for telephone support, telemonitoring, mHealth, eHealth self-management and RPM reviews. Therefore, repeated support from multiple reviews was interpreted as corroborative rather than fully independent. This judgement was used to temper the wording and interpretation of several statements.

### 3.4 Evidence summary

Twenty-seven practice-oriented statements were synthesised and organised into three tiers: essential core components, conditional implementation components, and emerging or outcome-related statements (Table 3). The source-basis column identifies whether statement specificity comes from outcome synthesis, intervention/component descriptions, implementation reasoning or cautious author interpretation, so that dose, alert and AI-related items are not presented as if they were supported by consistent subgroup analysis or meta-regression.

**Table 3:** Statement-level, tiered practice-oriented evidence statements for digital health education and self-management support in HF.

Tier/domain	#	Evidence statement	Source basis of specificity	Directness/certainty	How to use the statement
Essential core/organisation	1	Embed digital education within a nurse-led or multidisciplinary HF model when local staffing and service structure permit.	Review-level programme/component evidence; not a trial-tested mandate for a single staffing model.	Mostly direct HF evidence; quality mixed; overlap possible.	Use when workforce and service capacity can support ongoing review and follow-up.
Essential core/organisation	2	Assign clinical responsibility, review workflow and escalation rules whenever patient-generated data are transmitted.	Telemonitoring/RPM implementation features and safety rationale; exact response times were not tested consistently.	Direct telemonitoring/RPM evidence; operational specificity partly implementation-derived.	Use only with local protocols defining responsibility, timing and escalation.
Essential core/patient assessment	3	Assess digital literacy, device/network access, cognitive/sensory capacity, language needs, learning preferences and caregiver support before enrolment.	Consistent feasibility and implementation content across app, mHealth and distance-education reviews.	Direct and consistent implementation relevance; less dependent on a specific technology.	Use as a core feasibility/equity assessment before enrolment.
Essential core/patient assessment	4	Prioritise post-discharge or high-risk HF patients for digital education and follow-up when resources are limited.	Post-discharge and transitional-care review evidence; prioritisation is a pragmatic implementation decision.	Direct HF evidence, but intervention models and risk definitions vary.	Prioritise according to local workload, risk level and patient preference.
Essential core/modality selection	5	Select apps, messaging, web portals, telephone/IVR, wearables or RPM according to patient capacity, clinical purpose and infrastructure.	Intervention descriptions and comparative component synthesis; not evidence for one universally superior platform.	Direct and indirect evidence; mostly component/descriptive.	Select the platform according to patient capacity, clinical purpose and infrastructure.
Essential core/modality selection	6	Use feedback-enabled mHealth/RPM or structured support rather than stand-alone self-directed tools when clinical-service outcomes are targeted.	Effectiveness signal comes mainly from feedback-enabled STS/RPM/mHealth models; not from stand-alone education alone.	Direct HF evidence, but primary-study overlap likely.	Attribute outcomes to the feedback-enabled model, not to stand-alone tools.
Essential core/communication	7	Use synchronous contact, such as telephone coaching or video contact, as an optional complement to remote education and assessment.	Intervention descriptions and pooled telehealth/telecoaching evidence; contact frequency was not consistently tested.	Direct evidence with variable quality and dose.	Tailor synchronous contact to staffing capacity and patient need.
Essential core/usability	8	Design content and interfaces to be simple, accessible, culturally appropriate and usable for older adults with HF.	Usability/adaptation components described across mHealth/distance-education reviews.	Direct implementation relevance; outcome certainty variable.	Adapt to patient phenotype, literacy, accessibility needs and local practice.
Essential core/education content	9	Cover core HF self-management content: symptom recognition, daily weight, medication adherence, diet/fluid advice, activity, follow-up and when to seek help.	Common education content across HF self-care and digital education reviews; not a comparison of individual topics.	Direct HF relevance; content consistency relatively high.	Report delivered education topics and behavioural components clearly.
Essential core/behavioural support	10	Use theory-informed self-care strategies, goal setting and feedback where feasible.	Behaviour-change components and theory-informed programme descriptions; mechanism evidence is indirect.	Moderate directness; quality mixed.	Specify behaviour-change strategies and feedback mechanisms when reporting programmes.
Conditional implementation/app functions	11	When mobile apps are used, combine education with self-monitoring, reminders, feedback and communication functions where feasible.	Component evidence from app/mHealth reviews; no single bundle was isolated by meta-regression.	Direct mHealth evidence; methodological quality mixed.	Match app functions to patient burden, engagement capacity and available support.
Conditional implementation/monitoring	12	Include symptom, weight, blood pressure, pulse or activity monitoring only when data review and response capacity exist.	RPM/telemonitoring evidence supports monitoring with feedback; monitoring frequency varies by programme.	Direct evidence for feedback-enabled monitoring; daily frequency not consistently tested.	Use monitoring only when a response workflow is available.
Conditional implementation/alerts	13	Use alert thresholds or tiered risk rules as local protocol tools rather than universal evidence-based cut-offs.	Alert approaches are implementation features in RPM/telemonitoring; red-amber-green systems and 24-h response were not consistently tested.	Direct operational relevance but low certainty for exact thresholds.	Locally validate alert thresholds and review safety before use.
Conditional implementation/engagement	14	Use reminders, goal tracking and trend feedback to support engagement and self-management.	mHealth/app intervention components; isolated effects of each component are uncertain.	Direct but component-specific certainty limited.	Use reminders sparingly to support engagement without increasing notification burden.
Conditional implementation/RPM education	15	If RPM is implemented, integrate education and self-care coaching rather than treating monitoring alone as education.	Component synthesis from RPM/telemonitoring and self-management reviews.	Direct HF relevance with probable overlap.	Use RPM as monitoring plus education/feedback, not as education by itself.
Conditional implementation/asynchronous communication	16	Asynchronous messaging may support access, continuity and patient-provider communication, but should not replace urgent-care pathways.	Evidence partly from broader chronic-disease asynchronous communication reviews plus HF telehealth context.	Indirect for HF-specific education; quality variable.	Clarify response expectations, response times and urgent-care alternatives.
Conditional implementation/psychosocial support	17	Consider psychosocial support and reassurance within digital education, particularly for patients with anxiety, low confidence or high symptom burden.	Self-care and telehealth reviews describe psychosocial or coaching components; direct pooled effects vary.	Moderate directness; outcome measures heterogeneous.	Use as a supportive component and avoid overstating its independent effect.
Conditional implementation/dose and duration	18	Plan and report intervention duration, contact frequency and education intensity, but do not treat fixed thresholds as established requirements.	Intervention descriptions and narrative/component synthesis only; no consistent subgroup analysis or meta-regression supports 30-min sessions, > = 3 contacts/month, daily monitoring or > = 6 months as universal thresholds.	Direct descriptions but limited dose–response certainty.	Report dose and intensity; fixed thresholds are not recommendations.
Conditional implementation/hybrid care	19	Combine face-to-face education with digital reinforcement when patients need initial skills training, device support or complex discharge teaching.	Hybrid programme descriptions and post-discharge support evidence; comparative evidence is limited.	Direct but heterogeneous.	Use hybrid care for patients needing device support, initial skills training or complex discharge education.
Conditional implementation/equity	20	Tailor programmes for low literacy, rural residence, limited internet access, sensory/cognitive impairment and caregiver availability.	Equity and access considerations across digital health and rural HF reviews.	Direct implementation relevance; direct effectiveness evidence limited in vulnerable subgroups.	Build equity adaptations before scale-up.
Conditional implementation/governance	21	Address consent, privacy, data security, documentation, liability and professional accountability before implementation.	Safety/ethics/implementation rationale within reviews; not based on direct clinical outcome trials.	Indirect but highly applicable implementation requirement.	Implement only within institutional governance for privacy, liability and documentation.
Conditional implementation/safety escalation	22	Provide clear advice on red-flag symptoms, emergency escalation and the limits of digital communication.	Safety rationale plus telehealth/RPM implementation evidence; harm reporting remains limited.	Direct safety relevance; limited direct adverse-event evidence.	Ensure digital tools do not delay emergency care.
Emerging/AI tools	23	Treat AI or large-language-model tools as experimental adjuncts that require clinician oversight, local validation and safety boundaries.	Emerging methodological/observational evidence; no mature HF clinical-outcome evidence.	Early-stage and indirect.	Use only as an experimental adjunct with clinician oversight and local validation.
Emerging/expected outcomes	24	Feedback-enabled STS, RPM or mHealth may reduce HF-related hospitalisation in some analyses; do not generalise this to all digital education tools.	Outcome evidence belongs mainly to structured, feedback-enabled models.	Direct review-level evidence but overlap likely.	Stratify claims by intervention model and feedback loop.
Emerging/expected outcomes	25	Digital education and supported self-management may improve knowledge, self-care, self-efficacy and medication adherence, especially in structured or nurse-led models.	Outcome synthesis from digital education/eHealth self-management reviews.	Direct but measures and quality vary.	Interpret possible benefits cautiously by outcome, intervention model, engagement and methodological confidence; do not treat stand-alone education as equivalent to feedback-enabled care.
Emerging/expected outcomes	26	Quality of life may improve in some programmes, but effects differ by model, follow-up duration and measurement tool.	Heterogeneous outcome evidence across mHealth, eHealth and telemonitoring reviews.	Direct but inconsistent.	Avoid assuming uniform quality-of-life benefit across programmes.
Emerging/expected outcomes	27	Effects on all-cause hospitalisation and mortality are inconsistent and should be interpreted by intervention type, feedback loop and review quality.	Pooled findings differ across STS, telemonitoring/RPM and broader digital education reviews.	Direct evidence with heterogeneity and overlap.	Use as a cautionary effectiveness statement; avoid broad mortality or all-cause hospitalisation claims.

This table presents statement-level synthesis rather than review-level ratings. Review characteristics and AMSTAR 2 confidence are reported in Tables 1, 2. Each statement was developed by considering methodological quality, directness to adult HF digital education, consistency, applicability, feasibility and likely overlap among reviews. The final column explains how the statement should be applied or interpreted. These statements are evidence-informed implementation guidance, not formal clinical guideline recommendations or expert-consensus recommendations; no external expert panel was convened and no A/B recommendation grade was assigned.

## 4 Discussion

This best-evidence summary consolidates review-level evidence on digital education and digitally supported self-management for adults with HF. The synthesis identifies practice-oriented issues without assuming that all digital approaches have equivalent effects. Review-level evidence supports digital education as a complement to routine HF care, particularly when it is linked to clinical feedback, multidisciplinary follow-up and escalation pathways. However, the evidence base remains heterogeneous, includes indirect sources and is likely affected by overlap among reviews.

### 4.1 Interpreting effectiveness by intervention model

Effectiveness should be interpreted by intervention model rather than under a single broad label of digital health education. Structured telephone support, telemonitoring and RPM with professional feedback have the clearest review-level support for lower HF-related hospitalisation in some pooled analyses (9, 26, 27, 32). Nurse-led eHealth and telecoaching may support self-care and quality-of-life outcomes, but review quality and intervention intensity vary (23, 24). Mobile apps and mHealth education can support knowledge, reminders and self-management, particularly when they include monitoring, feedback and communication functions, but stand-alone apps or SMS reminders should not be assumed to produce the same hospitalisation or mortality effects as feedback-enabled RPM programmes (8, 10, 28, 29, 34). AI-assisted patient education remains early-stage and should not be presented as an established intervention model. The direct Cochrane review of mHealth education found no clear difference in HF knowledge and uncertain effects on self-efficacy, self-care and quality of life; more favourable signals arose mainly from broader or lower-confidence reviews (8).

### 4.2 Using evidence statements as conditional implementation guidance

Many implementation statements require local adaptation. Digital education is more likely to be feasible when it is integrated into an existing HF service, has a responsible clinical team and uses a clear feedback/escalation process. Nevertheless, resource-intensive components such as daily monitoring, rapid alert response, video contact or complex app platforms may be unrealistic in low-resource settings. For this reason, the evidence statements use conditional wording and distinguish examples from requirements. For example, intervention duration of at least 3 months or 6 months is treated as a reference range observed in several reviews rather than a universal threshold.

### 4.3 Equity, workforce burden and sustainability

Digital programmes may expand access, but they may also increase inequity when patients lack digital literacy, devices, connectivity, cognitive capacity or caregiver support. Older adults, frail patients, people with cognitive impairment, people with low literacy and rural patients may need simplified interfaces, caregiver involvement or low-technology approaches such as telephone or SMS. Implementation also creates workload for clinicians who must review patient-generated data, respond to alerts, provide education and maintain documentation. Cost-effectiveness, implementation fidelity, workforce sustainability and long-term maintenance were not consistently addressed in the included reviews and remain important research gaps.

### 4.4 AI and safety governance

AI and large-language-model tools should be considered emerging adjuncts rather than established HF education interventions. Current HF-related AI evidence largely concerns response accuracy, readability or methodological demonstrations, not clinical outcomes. AI-generated advice may be inaccurate, outdated, incomplete or inappropriate for medication titration, acute decompensation or emergency triage. Privacy, bias, legal accountability and professional responsibility also require careful governance. Therefore, AI tools should be used only with clinician oversight, local validation, clear limits of use and direct access to urgent clinical contact.

### 4.5 Limitations

This synthesis has limitations. First, the methodological quality of included reviews varied, and many reviews had low or critically low AMSTAR 2 ratings. Second, several sources were indirect because they included mixed chronic-disease populations, broad telehealth interventions or digital monitoring interventions rather than HF-specific digital education alone. Third, primary-study overlap across reviews was likely; because complete primary-study lists were not consistently available or extracted for all reviews, a formal corrected covered area analysis was not performed. Instead, likely overlap was considered qualitatively and reflected in downgraded statement strength. Fourth, the evidence statements were generated by the review authors and were not finalised through an external expert panel, so they should be interpreted as evidence-informed practice statements rather than formal consensus recommendations or clinical practice guideline recommendations. Fifth, the formal search was limited to Chinese and English bibliographic databases and did not include a systematic search of guideline repositories, professional organisation websites, JBI evidence summaries, WHO resources or grey literature; relevant guidance outside indexed bibliographic databases may therefore have been missed. Finally, evidence on cost-effectiveness, long-term sustainability, adverse events, HFpEF-specific populations, frail older adults, cognitive impairment, low literacy and low-resource settings remains limited.

### 4.6 Implications for practice and research

Clinical teams can use these statements as a structured reference when planning digital HF education, but implementation should begin with patient assessment, local workflow analysis and safety governance. Research should move beyond whether digital tools work in general and specify which components, intensity, feedback mechanisms and patient groups benefit. Future trials and implementation studies should report standardised outcomes, adverse events, cost-effectiveness, workforce burden, fidelity, acceptability, equity impact and long-term sustainability. Trials of AI-assisted education should include clinical validation, bias assessment, medication-safety safeguards and emergency-triage boundaries.

## 5 Conclusion

Review-level evidence suggests that digital education and digitally supported self-management may complement routine care for adults with HF, particularly when linked to structured clinical feedback, clearly assigned responsibility and escalation pathways. However, the evidence varies in methodological quality, directness and intervention type, and overlap among reviews is likely. The 27 evidence statements should therefore be interpreted as conditional, practice-oriented statements rather than formal guideline recommendations or uniformly high-certainty recommendations. Implementation should be adapted to patient capacity, digital literacy, local resources, workforce availability, privacy and safety requirements, and emergency-referral pathways.
