# Supplementary material for: Digital health education and self-management support for adults with heart failure: a best-evidence summary of review-level evidence
Source: Front Public Health. 2026 Sep 14;14:1926335. doi: 10.3389/fpubh.2026.1926335 (PMC13617182; doi:10.3389/fpubh.2026.1926335)
Supplement: Supplementary file 1 [file Table_1.DOCX]

**Supplementary Material S1. Full bibliographic database search strategies**

The bibliographic database search was conducted from database inception to 1 December 2025. Search strings were adapted to each database. The total number of records retrieved from the eight databases was 8,698. The search was limited to bibliographic databases; no systematic search of guideline repositories, professional society websites, JBI evidence summaries, WHO resources or grey literature was undertaken.

| **Source** | **Records** | **Search field / strategy** |
| --- | --- | --- |
| CNKI | 597 | TKA=("心力衰竭" + "心衰" + "慢性心力衰竭" + "心脏衰竭" + "心衰患者" + "心力衰竭管理") AND TKA=("平台" + "数字化" + "远程" + "互联网" + "数字" + "微信" + "软件" + "短信" + "智能" + "移动健康" + "mHealth" + "eHealth" + "应用程序" + "可穿戴" + "远程监测" + "人工智能" + "线上" + "在线") AND TKA=("健康教育" + "自我管理" + "患者教育" + "健康素养" + "自我效能" + "自我照顾" + "自我护理" + "用药依从性" + "体重监测" + "康复训练" + "延续性护理" + "生活质量") |
| Wanfang | 1,324 | 主题:("心力衰竭" OR "心衰" OR "慢性心力衰竭" OR "心脏衰竭" OR "心衰患者" OR "心力衰竭管理") AND 主题:("平台" OR "数字化" OR "远程" OR "互联网" OR "数字" OR "微信" OR "软件" OR "短信" OR "智能" OR "移动健康" OR "mHealth" OR "eHealth" OR "应用程序" OR "可穿戴" OR "远程监测" OR "人工智能" OR "线上" OR "在线") AND 主题:("健康教育" OR "自我管理" OR "患者教育" OR "健康素养" OR "自我效能" OR "自我照顾" OR "自我护理" OR "用药依从性" OR "体重监测" OR "康复训练" OR "延续性护理" OR "生活质量") |
| VIP/CQVIP | 852 | ("心力衰竭" OR "心衰" OR "慢性心力衰竭" OR "心脏衰竭" OR "心衰患者" OR "心力衰竭管理") AND ("平台" OR "数字化" OR "远程" OR "互联网" OR "数字" OR "微信" OR "软件" OR "短信" OR "智能" OR "移动健康" OR "mHealth" OR "eHealth" OR "应用程序" OR "可穿戴" OR "远程监测" OR "人工智能" OR "线上" OR "在线") AND ("健康教育" OR "自我管理" OR "患者教育" OR "健康素养" OR "自我效能" OR "自我照顾" OR "自我护理" OR "用药依从性" OR "体重监测" OR "康复训练" OR "延续性护理" OR "生活质量") |
| SinoMed | 2,569 | ("心力衰竭"[常用字段:智能] OR "心衰"[常用字段:智能] OR "慢性心力衰竭"[常用字段:智能] OR "心脏衰竭"[常用字段:智能] OR "心衰患者"[常用字段:智能] OR "心力衰竭管理"[常用字段:智能]) AND ("平台"[常用字段:智能] OR "数字化"[常用字段:智能] OR "远程"[常用字段:智能] OR "互联网"[常用字段:智能] OR "数字"[常用字段:智能] OR "微信"[常用字段:智能] OR "软件"[常用字段:智能] OR "短信"[常用字段:智能] OR "智能"[常用字段:智能] OR "移动健康"[常用字段:智能] OR "mHealth"[常用字段:智能] OR "eHealth"[常用字段:智能] OR "应用程序"[常用字段:智能] OR "可穿戴"[常用字段:智能] OR "远程监测"[常用字段:智能] OR "人工智能"[常用字段:智能] OR "线上"[常用字段:智能] OR "在线"[常用字段:智能]) AND ("健康教育"[常用字段:智能] OR "自我管理"[常用字段:智能] OR "患者教育"[常用字段:智能] OR "健康素养"[常用字段:智能] OR "自我效能"[常用字段:智能] OR "自我照顾"[常用字段:智能] OR "自我护理"[常用字段:智能] OR "用药依从性"[常用字段:智能] OR "体重监测"[常用字段:智能] OR "康复训练"[常用字段:智能] OR "延续性护理"[常用字段:智能] OR "生活质量"[常用字段:智能]) |
| Web of Science | 841 | TS=("heart failure" OR "cardiac failure" OR "chronic heart failure" OR "heart decompensation") AND TS=("platform" OR "digital" OR "digitization" OR "mHealth" OR "eHealth" OR "telemedicine" OR "remote monitoring" OR "internet" OR "wechat" OR "software" OR "text messag*" OR "smart" OR "intelligent" OR "ai" OR "Artificial Intelligence") AND TS=("patient education" OR "health education" OR "health literacy" OR "self-management" OR "self-care" OR "medication adherence" OR "self-efficacy") |
| PubMed | 781 | ("Heart Failure"[Mesh] OR "heart failure"[Title/Abstract] OR "cardiac failure"[Title/Abstract] OR "chronic heart failure"[Title/Abstract] OR "heart decompensation"[Title/Abstract]) AND ("Telemedicine"[Mesh] OR "platform"[Title/Abstract] OR "digital"[Title/Abstract] OR "digitization"[Title/Abstract] OR "mHealth"[Title/Abstract] OR "eHealth"[Title/Abstract] OR "telemedicine"[Title/Abstract] OR "remote monitoring"[Title/Abstract] OR "internet"[Title/Abstract] OR "wechat"[Title/Abstract] OR "software"[Title/Abstract] OR "text messag*"[Title/Abstract] OR "smart"[Title/Abstract] OR "intelligent"[Title/Abstract] OR "ai"[Title/Abstract] OR "Artificial Intelligence"[Title/Abstract]) AND ("Health Education"[Mesh] OR "patient education"[Title/Abstract] OR "health education"[Title/Abstract] OR "health literacy"[Title/Abstract] OR "self-management"[Title/Abstract] OR "self-care"[Title/Abstract] OR "medication adherence"[Title/Abstract] OR "self-efficacy"[Title/Abstract]) |
| Cochrane Library | 708 | ("heart failure" OR "cardiac failure" OR "chronic heart failure" OR "heart decompensation") AND ("digital" OR "mHealth" OR "eHealth" OR "telemedicine" OR "remote monitoring" OR "internet" OR "software" OR "text messaging" OR "smart" OR "artificial intelligence") AND ("patient education" OR "health education" OR "self-management" OR "self-care" OR "medication adherence" OR "self-efficacy") |
| Embase | 1,026 | ('heart failure':ti,ab,kw OR 'cardiac failure':ti,ab,kw OR 'chronic heart failure':ti,ab,kw OR 'heart decompensation':ti,ab,kw) AND ('platform':ti,ab,kw OR 'digitization':ti,ab,kw OR 'mhealth':ti,ab,kw OR 'ehealth':ti,ab,kw OR 'telemedicine':ti,ab,kw OR 'remote monitoring':ti,ab,kw OR 'digital':ti,ab,kw OR 'internet':ti,ab,kw OR 'wechat':ti,ab,kw OR 'software':ti,ab,kw OR 'text messag*':ti,ab,kw OR 'smart':ti,ab,kw OR 'intelligent':ti,ab,kw OR 'ai':ti,ab,kw OR 'artificial intelligence':ti,ab,kw) AND ('patient education':ti,ab,kw OR 'health education':ti,ab,kw OR 'health literacy':ti,ab,kw OR 'self-management':ti,ab,kw OR 'self-care':ti,ab,kw OR 'medication adherence':ti,ab,kw OR 'self-efficacy':ti,ab,kw) |

Reports available online by the search cutoff were eligible even if assigned to a later print issue.
