# Supplementary material for: Digital health education and self-management support for adults with heart failure: a best-evidence summary of review-level evidence
Source: Front Public Health. 2026 Sep 14;14:1926335. doi: 10.3389/fpubh.2026.1926335 (PMC13617182; doi:10.3389/fpubh.2026.1926335)
Supplement: Supplementary file 2 [file Table_2.DOCX]

**Supplementary Material S2. AMSTAR 2 item-level appraisal matrix and scoping-review appraisal notes**

Code definitions: Y = yes; PY = partial yes; N = no; NA = not applicable; NR = not reported or not sufficiently reported. Each AMSTAR 2 item is assigned one final conservative code. When the available information did not support a definitive positive judgement, PY or NR was assigned rather than combining multiple codes. Overall confidence followed AMSTAR 2 rules: one critical flaw was rated low and more than one critical flaw was rated critically low. For this conservative overall judgement, NR in a critical domain was treated as not meeting the criterion.

Systematic reviews, meta-analyses, the network meta-analysis, umbrella review and systematic metareview were appraised with AMSTAR 2. Scoping reviews were not rated with AMSTAR 2 and were assessed descriptively using PRISMA-ScR/JBI-informed criteria. The matrix uses the following abbreviations: Y = yes; PY = partial yes; N = no; NA = not applicable; NR = not reported/unclear. Where the available appraisal documentation did not support a definitive positive judgement, the item was recorded conservatively as NR or PY rather than assumed to be yes.

**Table S2A. AMSTAR 2 items 1-8**

| **Ref.** | **Overall** | **I1 PICO/components** | **I2 protocol** | **I3 design rationale** | **I4 search** | **I5 duplicate selection** | **I6 duplicate extraction** | **I7 excluded list** | **I8 included-study description** |
| --- | --- | --- | --- | --- | --- | --- | --- | --- | --- |
| 7 | High | Y | Y | Y | Y | Y | Y | PY | Y |
| 8 | High | Y | Y | Y | Y | Y | Y | PY | Y |
| 9 | High | Y | Y | Y | Y | Y | Y | PY | Y |
| 10 | Low | Y | PY | PY | Y | PY | PY | N | Y |
| 11 | Critically low | PY | NR | PY | PY | PY | PY | N | PY |
| 12 | Low | Y | PY | PY | Y | PY | PY | N | Y |
| 13 | Critically low | PY | NR | PY | PY | PY | PY | N | PY |
| 14 | Critically low | PY | NR | PY | PY | PY | PY | N | PY |
| 15 | Low | Y | PY | PY | Y | PY | PY | N | Y |
| 18 | Critically low | PY | NR | PY | PY | PY | NR | N | PY |
| 19 | Critically low | PY | NR | PY | PY | PY | NR | N | PY |
| 20 | Critically low | PY | NR | PY | PY | PY | PY | N | PY |
| 21 | Critically low | PY | NR | PY | PY | PY | NR | N | PY |
| 22 | Critically low | PY | NR | PY | PY | PY | PY | N | PY |
| 23 | Critically low | PY | NR | PY | PY | PY | PY | N | PY |
| 24 | Low | Y | PY | PY | Y | PY | PY | N | Y |
| 25 | Critically low | PY | NR | PY | PY | PY | PY | N | PY |
| 26 | Low | Y | PY | PY | Y | PY | PY | N | Y |
| 27 | Critically low | PY | NR | PY | PY | PY | PY | N | PY |
| 28 | Critically low | PY | NR | PY | PY | PY | PY | N | PY |
| 29 | Critically low | PY | NR | PY | PY | PY | PY | N | PY |
| 30 | Critically low | PY | NR | PY | PY | PY | NR | N | PY |
| 31 | Critically low | PY | NR | PY | PY | PY | PY | N | PY |
| 32 | Critically low | PY | NR | PY | PY | PY | NR | N | PY |
| 33 | Critically low | PY | NR | PY | PY | PY | PY | N | PY |

**Table S2B. AMSTAR 2 items 9-16 and use in synthesis**

| **Ref.** | **Overall** | **I9 RoB tool** | **I10 funding of primary studies** | **I11 meta-analysis methods** | **I12 RoB impact on results** | **I13 RoB in interpretation** | **I14 heterogeneity** | **I15 publication bias** | **I16 review COI/funding** | **Key concerns/use in synthesis** |
| --- | --- | --- | --- | --- | --- | --- | --- | --- | --- | --- |
| 7 | High | Y | PY | Y | Y | Y | Y | PY | Y | High-confidence review; interpreted according to directness and overlap. |
| 8 | High | Y | PY | Y | Y | Y | Y | PY | Y | High-confidence review; interpreted according to directness and overlap. |
| 9 | High | Y | PY | Y | Y | Y | Y | PY | Y | High-confidence review; interpreted according to directness and overlap. |
| 10 | Low | PY | NR | PY | PY | PY | PY | PY | PY | One critical flaw; used cautiously and not as the sole basis for a strong practice statement. |
| 11 | Critically low | PY | NR | PY | PY | PY | PY | PY | PY | More than one critical flaw; used only as contextual/supportive evidence and not as the sole basis for a strong practice statement. |
| 12 | Low | PY | NR | PY | PY | PY | PY | PY | PY | One critical flaw; used cautiously and not as the sole basis for a strong practice statement. |
| 13 | Critically low | PY | NR | PY | PY | PY | PY | PY | PY | More than one critical flaw; used only as contextual/supportive evidence and not as the sole basis for a strong practice statement. |
| 14 | Critically low | PY | NR | PY | PY | PY | PY | PY | PY | More than one critical flaw; used only as contextual/supportive evidence and not as the sole basis for a strong practice statement. |
| 15 | Low | PY | NR | PY | PY | PY | PY | PY | PY | One critical flaw; used cautiously and not as the sole basis for a strong practice statement. |
| 18 | Critically low | PY | NR | PY | N | PY | PY | N | PY | More than one critical flaw; used only as contextual/supportive evidence and not as the sole basis for a strong practice statement. |
| 19 | Critically low | PY | NR | PY | N | PY | PY | N | PY | More than one critical flaw; used only as contextual/supportive evidence and not as the sole basis for a strong practice statement. |
| 20 | Critically low | PY | NR | PY | PY | PY | PY | PY | PY | More than one critical flaw; used only as contextual/supportive evidence and not as the sole basis for a strong practice statement. |
| 21 | Critically low | PY | NR | PY | N | PY | PY | N | PY | More than one critical flaw; used only as contextual/supportive evidence and not as the sole basis for a strong practice statement. |
| 22 | Critically low | PY | NR | PY | PY | PY | PY | PY | PY | More than one critical flaw; used only as contextual/supportive evidence and not as the sole basis for a strong practice statement. |
| 23 | Critically low | PY | NR | PY | PY | PY | PY | PY | PY | More than one critical flaw; used only as contextual/supportive evidence and not as the sole basis for a strong practice statement. |
| 24 | Low | PY | NR | PY | PY | PY | PY | PY | PY | One critical flaw; used cautiously and not as the sole basis for a strong practice statement. |
| 25 | Critically low | PY | NR | PY | PY | PY | PY | PY | PY | More than one critical flaw; used only as contextual/supportive evidence and not as the sole basis for a strong practice statement. |
| 26 | Low | PY | NR | PY | PY | PY | PY | PY | PY | One critical flaw; used cautiously and not as the sole basis for a strong practice statement. |
| 27 | Critically low | PY | NR | PY | PY | PY | PY | PY | PY | More than one critical flaw; used only as contextual/supportive evidence and not as the sole basis for a strong practice statement. |
| 28 | Critically low | PY | NR | PY | PY | PY | PY | PY | PY | More than one critical flaw; used only as contextual/supportive evidence and not as the sole basis for a strong practice statement. |
| 29 | Critically low | PY | NR | PY | PY | PY | PY | PY | PY | More than one critical flaw; used only as contextual/supportive evidence and not as the sole basis for a strong practice statement. |
| 30 | Critically low | PY | NR | PY | N | PY | PY | N | PY | More than one critical flaw; used only as contextual/supportive evidence and not as the sole basis for a strong practice statement. |
| 31 | Critically low | PY | NR | PY | PY | PY | PY | PY | PY | More than one critical flaw; used only as contextual/supportive evidence and not as the sole basis for a strong practice statement. |
| 32 | Critically low | PY | NR | PY | N | PY | PY | N | PY | More than one critical flaw; used only as contextual/supportive evidence and not as the sole basis for a strong practice statement. |
| 33 | Critically low | PY | NR | PY | PY | PY | PY | PY | PY | More than one critical flaw; used only as contextual/supportive evidence and not as the sole basis for a strong practice statement. |

**Table S2C. Scoping-review appraisal notes**

| **Ref.** | **Approach** | **Reporting/appraisal basis** | **Key concerns** | **Use in synthesis** |
| --- | --- | --- | --- | --- |
| 16 | Scoping review | PRISMA-ScR/JBI-informed descriptive assessment | Mapping evidence; not designed for AMSTAR 2 or causal-effect certainty grading. | Used for implementation context and domain mapping; not used as sole basis for strong effectiveness claims. |
| 17 | Scoping review | PRISMA-ScR/JBI-informed descriptive assessment | Mapping evidence; not designed for AMSTAR 2 or causal-effect certainty grading. | Used for implementation context and domain mapping; not used as sole basis for strong effectiveness claims. |
