# Supplementary material for: Digital health education and self-management support for adults with heart failure: a best-evidence summary of review-level evidence
Source: Front Public Health. 2026 Sep 14;14:1926335. doi: 10.3389/fpubh.2026.1926335 (PMC13617182; doi:10.3389/fpubh.2026.1926335)
Supplement: Supplementary file 3 [file Table_3.DOCX]

**Supplementary Material S3. Evidence map, directness, overlap risk and source basis of specificity**

This evidence map links each practice-oriented statement to supporting review-level evidence and makes explicit whether statement specificity was based on outcome synthesis, intervention/component descriptions, implementation reasoning or cautious author interpretation. It is not a corrected covered area analysis. Likely overlap was judged qualitatively: high indicates the same review lineage or multiple reviews of the same modality and time window likely sharing core trials; moderate indicates related populations, interventions or time windows with probable partial overlap; low indicates substantively distinct evidence topics; and not applicable indicates a single supporting review. Overlap and directness were used to temper statement wording. The final column corresponds exactly to Table 3 and explains how each statement should be applied or interpreted; it is not a recommendation grade.

| **#** | **Tier** | **Statement short label** | **Supporting refs** | **Source basis of specificity** | **Directness** | **Likely overlap risk** | **How to use the statement** |
| --- | --- | --- | --- | --- | --- | --- | --- |
| 1 | Essential core | Nurse-led/multidisciplinary model | 12,16,20,21,22 | Review-level programme/component evidence; not a trial-tested mandate for a single staffing model. | Mostly direct HF evidence; quality mixed; overlap possible. | Moderate | use when workforce and service capacity can support ongoing review and follow-up. |
| 2 | Essential core | Clinical responsibility and escalation | 7,9,23,24 | Telemonitoring/RPM implementation features and safety rationale; exact response times were not tested consistently. | Direct telemonitoring/RPM evidence; operational specificity partly implementation-derived. | High | use only with local protocols defining responsibility, timing and escalation. |
| 3 | Essential core | Digital literacy and access assessment | 10,23,25,26,27 | Consistent feasibility and implementation content across app, mHealth and distance-education reviews. | Direct and consistent implementation relevance; less dependent on a specific technology. | High | use as a core feasibility/equity assessment before enrolment. |
| 4 | Essential core | Post-discharge/high-risk prioritisation | 15,17,21,28 | Post-discharge and transitional-care review evidence; prioritisation is a pragmatic implementation decision. | Direct HF evidence, but intervention models and risk definitions vary. | Moderate | prioritise according to local workload, risk level and patient preference. |
| 5 | Essential core | Platform selection by patient/context | 10,16,23,25,26 | Intervention descriptions and comparative component synthesis; not evidence for one universally superior platform. | Direct and indirect evidence; mostly component/descriptive. | High | select the platform according to patient capacity, clinical purpose and infrastructure. |
| 6 | Essential core | Feedback-enabled models for service outcomes | 7,9,23,25,29 | Effectiveness signal comes mainly from feedback-enabled STS/RPM/mHealth models; not from stand-alone education alone. | Direct HF evidence, but primary-study overlap likely. | High | attribute outcomes to the feedback-enabled model, not to stand-alone tools. |
| 7 | Essential core | Synchronous contact as optional support | 9,13,21 | Intervention descriptions and pooled telehealth/telecoaching evidence; contact frequency was not consistently tested. | Direct evidence with variable quality and dose. | Moderate | tailor synchronous contact to staffing capacity and patient need. |
| 8 | Essential core | Usable and accessible content/interface | 10,16,26,27 | Usability/adaptation components described across mHealth/distance-education reviews. | Direct implementation relevance; outcome certainty variable. | Moderate | adapt to patient phenotype, literacy, accessibility needs and local practice. |
| 9 | Essential core | Core HF self-management content | 8,27,31 | Common education content across HF self-care and digital education reviews; not a comparison of individual topics. | Direct HF relevance; content consistency relatively high. | Moderate | report delivered education topics and behavioural components clearly. |
| 10 | Essential core | Theory-informed behavioural support | 12,20,28 | Behaviour-change components and theory-informed programme descriptions; mechanism evidence is indirect. | Moderate directness; quality mixed. | Moderate | specify behaviour-change strategies and feedback mechanisms when reporting programmes. |
| 11 | Conditional implementation | App functions and communication | 10,25,26 | Component evidence from app/mHealth reviews; no single bundle was isolated by meta-regression. | Direct mHealth evidence; methodological quality mixed. | High | match app functions to patient burden, engagement capacity and available support. |
| 12 | Conditional implementation | Monitoring with response capacity | 9,23,29 | RPM/telemonitoring evidence supports monitoring with feedback; monitoring frequency varies by programme. | Direct evidence for feedback-enabled monitoring; daily frequency not consistently tested. | High | use monitoring only when a response workflow is available. |
| 13 | Conditional implementation | Local alert thresholds/risk rules | 9,23,24 | Alert approaches are implementation features in RPM/telemonitoring; red-amber-green systems and 24-hour response were not consistently tested. | Direct operational relevance but low certainty for exact thresholds. | High | locally validate alert thresholds and review safety before use. |
| 14 | Conditional implementation | Reminders, goals and trend feedback | 10,25,26 | mHealth/app intervention components; isolated effects of each component are uncertain. | Direct but component-specific certainty limited. | High | use reminders sparingly to support engagement without increasing notification burden. |
| 15 | Conditional implementation | RPM with education/coaching | 9,23,29 | Component synthesis from RPM/telemonitoring and self-management reviews. | Direct HF relevance with probable overlap. | High | use RPM as monitoring plus education/feedback, not as education by itself. |
| 16 | Conditional implementation | Asynchronous communication boundaries | 17,32 | Evidence partly from broader chronic-disease asynchronous communication reviews plus HF telehealth context. | Indirect for HF-specific education; quality variable. | Low | clarify response expectations, response times and urgent-care alternatives. |
| 17 | Conditional implementation | Psychosocial support/reassurance | 12,20,21 | Self-care and telehealth reviews describe psychosocial or coaching components; direct pooled effects vary. | Moderate directness; outcome measures heterogeneous. | Moderate | use as a supportive component and avoid overstating its independent effect. |
| 18 | Conditional implementation | Dose/duration reporting without fixed thresholds | 8,21,27,28 | Intervention descriptions and narrative/component synthesis only; no consistent subgroup analysis or meta-regression supports 30-minute sessions, >=3 contacts/month, daily monitoring or >=6 months as universal thresholds. | Direct descriptions but limited dose-response certainty. | Moderate | report dose and intensity; fixed thresholds are not recommendations. |
| 19 | Conditional implementation | Hybrid face-to-face plus digital support | 15,17,20 | Hybrid programme descriptions and post-discharge support evidence; comparative evidence is limited. | Direct but heterogeneous. | Moderate | use hybrid care for patients needing device support, initial skills training or complex discharge education. |
| 20 | Conditional implementation | Equity adaptations | 27,33 | Equity and access considerations across digital health and rural HF reviews. | Direct implementation relevance; direct effectiveness evidence limited in vulnerable subgroups. | Low | build equity adaptations before scale-up. |
| 21 | Conditional implementation | Privacy, governance and accountability | 14,23,32 | Safety/ethics/implementation rationale within reviews; not based on direct clinical outcome trials. | Indirect but highly applicable implementation requirement. | Low | implement only within institutional governance for privacy, liability and documentation. |
| 22 | Conditional implementation | Red-flag symptoms and emergency escalation | 9,23,24 | Safety rationale plus telehealth/RPM implementation evidence; harm reporting remains limited. | Direct safety relevance; limited direct adverse-event evidence. | High | ensure digital tools do not delay emergency care. |
| 23 | Emerging | AI/LLM tools as experimental adjuncts | 14 | Emerging methodological/observational evidence; no mature HF clinical-outcome evidence. | Early-stage and indirect. | Not applicable | use only as an experimental adjunct with clinician oversight and local validation. |
| 24 | Emerging | HF-related hospitalisation claims by model | 7,9,23,29 | Outcome evidence belongs mainly to structured, feedback-enabled models. | Direct review-level evidence but overlap likely. | High | stratify claims by intervention model and feedback loop. |
| 25 | Emerging | Knowledge, self-care and adherence outcomes | 8,20,27,28 | Outcome synthesis from digital education/eHealth self-management reviews. | Direct but measures and quality vary. | High | Interpret possible benefits cautiously by outcome, intervention model, engagement and methodological confidence; do not treat stand-alone education as equivalent to feedback-enabled care. |
| 26 | Emerging | Quality-of-life outcomes | 10,25,26,31 | Heterogeneous outcome evidence across mHealth, eHealth and telemonitoring reviews. | Direct but inconsistent. | High | avoid assuming uniform quality-of-life benefit across programmes. |
| 27 | Emerging | All-cause hospitalisation/mortality caution | 7,9,29 | Pooled findings differ across STS, telemonitoring/RPM and broader digital education reviews. | Direct evidence with heterogeneity and overlap. | High | use as a cautionary effectiveness statement; avoid broad mortality or all-cause hospitalisation claims. |
