# Supplementary material for: Digital health education and self-management support for adults with heart failure: a best-evidence summary of review-level evidence
Source: Front Public Health. 2026 Sep 14;14:1926335. doi: 10.3389/fpubh.2026.1926335 (PMC13617182; doi:10.3389/fpubh.2026.1926335)
Supplement: Supplementary file 4 [file Table_4.DOCX]

**Supplementary Material S4. PRISMA 2020 checklist**

| **Section/topic** | **Item** | **Checklist requirement** | **Location in manuscript** |
| --- | --- | --- | --- |
| Title | 1 | Identify the report as a systematic review. | Title page |
| Abstract | 2 | Provide a structured summary. | Abstract |
| Rationale | 3 | Describe the rationale in context. | Introduction |
| Objectives | 4 | Provide an explicit statement of objectives. | Introduction |
| Eligibility criteria | 5 | Specify inclusion/exclusion criteria and grouping for syntheses. | Methods 2.2 and 2.4 |
| Information sources | 6 | Specify all sources and the date last searched. | Methods 2.3; Supplementary Material S1 |
| Search strategy | 7 | Present the full search strategies. | Supplementary Material S1 |
| Selection process | 8 | Specify methods used to decide eligibility. | Methods 2.5; Figure 1 |
| Data collection process | 9 | Specify methods used to collect data. | Methods 2.8 |
| Data items | 10a | List and define outcomes sought. | Methods 2.2 and 2.8 |
| Data items | 10b | List and define other variables sought and assumptions. | Methods 2.2 and 2.8 |
| Risk of bias | 11 | Specify appraisal methods and tools. | Methods 2.6; Supplementary Material S2 |
| Effect measures | 12 | Specify effect measures for each outcome. | Not applicable; no quantitative pooling was conducted |
| Synthesis methods | 13a | Describe eligibility for each synthesis. | Methods 2.2, 2.4 and 2.8 |
| Synthesis methods | 13b | Describe data preparation. | Methods 2.8 |
| Synthesis methods | 13c | Describe methods used to tabulate or visually display results. | Methods 2.8; Tables 1-3; Supplementary Material S3 |
| Synthesis methods | 13d | Describe methods used to synthesise results and rationale. | Methods 2.1 and 2.8 |
| Synthesis methods | 13e | Describe methods used to explore heterogeneity. | Narrative comparison by intervention model, directness and methodological confidence; no meta-regression |
| Synthesis methods | 13f | Describe sensitivity analyses. | Not applicable; no quantitative pooling or sensitivity analysis |
| Reporting bias methods | 14 | Describe methods used to assess risk of bias due to missing results. | Publication-bias reporting was captured within AMSTAR 2; no separate cross-review assessment |
| Certainty assessment | 15 | Describe methods used to assess certainty/confidence. | Methods 2.6-2.8; qualitative interpretation using appraisal, directness, consistency, applicability and overlap |
| Study selection | 16a | Describe search and selection results. | Results 3.1; Figure 1 |
| Study selection | 16b | Cite excluded reports that appeared eligible and explain exclusion. | Supplementary Material S5 |
| Study characteristics | 17 | Cite each included study and present its characteristics. | Table 1 |
| Risk of bias | 18 | Present appraisal results for each included study. | Table 1; Table 2; Supplementary Material S2 |
| Individual results | 19 | Present results for all outcomes for each study. | Table 1; Table 3; Supplementary Material S3 |
| Syntheses | 20a | Summarise characteristics and appraisal for each synthesis. | Results 3.2-3.4; Tables 1-3 |
| Syntheses | 20b | Present results of statistical syntheses. | Not applicable; no new meta-analysis |
| Syntheses | 20c | Present investigations of heterogeneity. | Narrative interpretation by intervention model, directness, quality and overlap |
| Syntheses | 20d | Present sensitivity analyses. | Not applicable; no quantitative sensitivity analysis |
| Reporting biases | 21 | Present assessments of reporting bias. | AMSTAR 2 item-level results in Supplementary Material S2; no separate assessment |
| Certainty of evidence | 22 | Present certainty/confidence assessments. | Results 3.2-3.4; Table 3; Supplementary Material S3 |
| Discussion | 23a | Provide a general interpretation in context. | Discussion 4.1-4.3 |
| Discussion | 23b | Discuss limitations of the included evidence. | Discussion 4.4-4.5 |
| Discussion | 23c | Discuss limitations of the review process. | Discussion 4.5 |
| Discussion | 23d | Discuss implications for practice and research. | Discussion 4.2-4.6; Conclusion |
| Registration and protocol | 24a | Provide registration information. | Methods 2.1 |
| Registration and protocol | 24b | Indicate where the protocol can be accessed. | Not reported |
| Registration and protocol | 24c | Describe amendments to registration or protocol. | Not reported |
| Support | 25 | Describe financial or non-financial support. | Funding |
| Competing interests | 26 | Declare competing interests. | Conflict of interest |
| Availability | 27 | Report availability of data, code and materials. | Data availability statement; Supplementary Materials S1-S5 |
