# Supplementary material for: Digital health education and self-management support for adults with heart failure: a best-evidence summary of review-level evidence
Source: Front Public Health. 2026 Sep 14;14:1926335. doi: 10.3389/fpubh.2026.1926335 (PMC13617182; doi:10.3389/fpubh.2026.1926335)
Supplement: Supplementary file 5 [file Table_5.DOCX]

**Supplementary Material S5. Excluded full-text reports with reasons**

This table lists the ten reports excluded after full-text assessment. Exclusion categories and totals correspond directly to Figure 1.

| **Stage** | **Source ID** | **Title** | **Year/source** | **Reason for exclusion** |
| --- | --- | --- | --- | --- |
| Full-text assessment | 3830 | Effectiveness of mobile health technology interventions on patients with heart failure: Systematic review and meta-analysis | 2019, Circulation supplement | Conference abstract or abstract-only supplement; duplicated or incomplete report of a later full review. Not treated as an independent full systematic evidence synthesis. |
| Full-text assessment | 3839 | Effectiveness of technology based education on quality of life in patients with congestive heart failure | 2021, Cardiopulmonary Physical Therapy Journal | Abstract/short-report record with insufficient methodological and full-text details for final inclusion. |
| Full-text assessment | 4784 | Remote (non-invasive) monitoring in heart failure: Effect on length of stay, quality of life, knowledge, adherence and satisfaction in 8,323 heart failure patients: A systematic review | 2010, European Heart Journal supplement | Conference abstract or less complete report overlapping with a complete review; not treated as an independent full report. |
| Full-text assessment | 4817 | Results from a systematic review and meta-analysis of remote (non-invasive) monitoring in 8,323 heart failure patients on length of stay, quality of life, knowledge, compliance and satisfaction | 2010, European Journal of Heart Failure supplement | Conference abstract or less complete report overlapping with a complete review; not treated as an independent full report. |
| Full-text assessment | 5026 | Telemonitoring as a factor in the adherence to self-care in the person with Heart Failure | 2023, conference abstract | Conference abstract with insufficient full systematic-review details for inclusion. |
| Full-text assessment | 5118 | The effectiveness of telehealth nursing in heart failure self-management | 2017, conference abstract | Conference abstract with insufficient full systematic-review details for inclusion. |
| Full-text assessment | 5208 | Transitional Care Interventions To Prevent Readmissions for People With Heart Failure | 2014, evidence report/book record | Not relevant to the review intervention/content: broad transitional-care evidence not specific to digital health education or digitally supported self-management. |
| Full-text assessment | 5268 | Using mHealth to reduce heart failure related in-hospital care - a meta-analysis | 2022, conference abstract | Conference abstract with insufficient full report details and potential overlap with included mHealth/RPM reviews. |
| Full-text assessment | 4839 | Scoping review of transformative interventions, using validated quality of life assessment tools in heart failure (HF) patients with depression | 2023, conference/abstract record | Conference/abstract-only record with insufficient full systematic-review methods and report detail for inclusion. |
| Full-text assessment | 4217 | Impact of Telehealth on Patient Self-management of Heart Failure: A Review of Literature | 2012, review article | Narrative/literature review rather than a systematic review-level evidence synthesis; no formal review protocol, risk-of-bias appraisal or quantitative synthesis was identifiable from the record. |
